# White coat hypertension in acute retinal vein occlusion

**DOI:** 10.1186/s40942-024-00584-y

**Published:** 2024-09-18

**Authors:** Shuichiro Aoki, Haruyuki Suzuki, Kohei Ueda, Kohdai Kitamoto, Keiko Azuma, Ryo Obata

**Affiliations:** 1 Department of Ophthalmology, the University of Tokyo Graduate School of Medicine, 7-3-1 Hongo, Bunkyo-ku, Tokyo, 113-8655 Japan; 2 Department of Ophthalmology, Toranomon Hospital, 2-2-2, Toranomon, Minato-ku, 105-8470 Tokyo, Japan

**Keywords:** White-coat hypertension, Blood pressure, Retinal vein occlusion, Retinal vascular disease

## Abstract

**Purpose:**

To investigate the association between white-coat hypertension (WCH) and acute retinal vein occlusion (RVO).

**Methods:**

In this retrospective case-control study, patients aged 40 years or older diagnosed with acute-phase RVO were included. Patients with other pathologies served as non-RVO controls. Blood pressure (BP) was measured in the office during their initial visit, and information about home BP and hypertension (HTN) medication was obtained through interviews. After 1:2 age and sex-matching between the RVO and non-RVO groups, the proportions of HTN cases were compared. A similar comparison was made in subgroups with or without HTN medication.

**Results:**

Fifty-one patients with RVO and 102 with non-RVO were included in the analysis. For the entire cohort, the RVO group exhibited a significantly greater proportion of WCH and sustained HTN compared to the non-RVO group. In the subgroup without HTN treatment, the proportion of WCH or sustained HTN was still significantly higher in the RVO group. However, in the subgroup receiving HTN treatment, the proportion of WCH or sustained HTN was higher in the RVO group than in the non-RVO group, though not statistically significant.

**Conclusion:**

This case-control study suggests that WCH may be associated with RVO, particularly in patients without HTN treatment. Given that interventions for WCH have not been standardized, a more detailed and prospective study is warranted to elucidate the risk of WCH for RVO and other retinal vascular diseases.

## Introduction

Hypertension (HTN) is a typical risk factor for retinal vein occlusion (RVO) [1]. Appropriate blood pressure (BP) control in RVO patients is important for the prognosis of visual function [2–4]. The measurement of BP in the office is the basis for the diagnosis and follow-up of hypertension [5]. However, home BP or ambulatory BP measurements are more closely associated with hypertension-induced organ damage and the risk of cardiovascular events, leading to the recommendation that home BP should be used as an indicator of BP control [5, 6]. Based on the office BP and the home BP, patients are classified into four categories: sustained HTN (elevated office BP and elevated home BP), masked HTN, white-coat HTN, and normal BP [6, 7]. Masked HTN is a state where office BP is normal, but home BP is high. The cardiovascular risk of untreated masked HTN is reported to be comparable to that of sustained HTN [6, 8].

On the other hand, white coat hypertension (WCH) is a state where office BP is high, but home BP is normal. The similar state of the patients with HTN medication is commonly called treated white-coat effect (WCE) or white-coat uncontrolled hypertension (WCUH) [9]. White-coat HTN is reproducible phenomenon, and it occurs in 10 to 30% of patients with elevated office BP [10, 11]. Anxiety or the sympathetic nervous system may be a cause of WCH [11]. In the past, WCH or WCUH was not actively treated because studies have shown no or a weak increase in cardiovascular risks or mortality [12–14]. However, more recent research has suggested that WCH may indeed be linked to targeted organ damage and increased mortality [15, 16]. This evolving understanding raises the possibility that WCH could also be a risk factor for developing RVO. Despite this, our literature review did not find any studies directly examining the relationship between WCH and RVO, indicating a gap in current knowledge.

Therefore, this study aims to test the hypothesis that RVO patients may have a higher prevalence of WCH or WCUH than non-RVO patients.

## Methods

### Study design

This retrospective case-control study was designed to evaluate WCH or WCUH in acute RVO. It was conducted according to the tenets of the Declaration of Helsinki and approved by the institutional review board (IRB) of the University of Tokyo. Written informed consent was not required from the IRB. However, participants who did not grant authorization to use their medical records for research were excluded from the study.

### Patients

We reviewed the charts of patients who visited the medical retina outpatient clinic of our department for the first time from August 2021 to April 2023. All patients underwent visual acuity testing, slit-lamp biomicroscopy, fundoscopy, optical coherence tomography (OCT). OCT angiography and/or fluorescein angiography (FA) were performed if indicated. Diagnosis of RVO was made by experienced retina specialist, based mainly on the fundoscopic findings such as venous dilation, retinal edema, intra-retinal hemorrhages, or cotton wool spots [1]. The results of OCT, OCTA, FA, or other modalities such as indocyanine-green angiography were referred to support the diagnosis or to rule out differential diagnosis. As for cases without RVO, routine examination and the ancillary tests based on the differential diagnosis were performed to make a diagnosis. Office BP, home BP, current hypertension treatment, and history of diabetes and hyperlipidemia, as recorded during the visit, were reviewed for the study.

### BP measurement and patient classification

In our department, routine BP measurement was conducted at the first visit for each patient. Blood pressure was measured twice using an automatic upper arm sphygmomanometer available in the outpatient clinic. During the measurement, patients were instructed to remain seated and at rest. The average of the two measurements was recorded as the office BP. If it was 140/90 or higher, BP was measured again by auscultation by medical staff, and this value was recorded as the office BP. According to Hypertension Treatment Guidelines in Japan [6], elevated office BP was defined as over 140 mmHg and/or 90 mmHg for systolic and diastolic pressure, respectively and elevated home BP was defined as over 135 mmHg and/or 85 mmHg for systolic and diastolic pressure, respectively. Based on these criteria, office BP phenotypes were categorized as normal office BP and elevated office BP. On the same visit, each patient had been asked about their BP measurements at home (home BP). Both office BP and home BP were retrospectively collected from medical charts.

Exclusion criteria was as follows. As the aim of our study was to examine relationship between WCH and RVO, patients were excluded if the medical chart did not document office BP or documented elevated office BP without home BP. Patients with both elevated office BP and home BP was classified as having sustained hypertension (if not undergoing HTN treatment) or uncontrolled hypertension (if undergoing HTN treatment); those with elevated office BP and normal home BP were classified as WCH (if not on HTN treatment) WCUH (if on HTN treatment) [9].

In our preliminary retrospective review, most patients with normal office BP did not have home BP documented, because they did not have a BP recorder at home. To ensure a sufficient sample size for the control group with normal office BP, these patients were included regardless of availability of home BP but were not further sub-categorized.

Patients without documentation of HTN medication were also excluded from the study, as this information is essential for evaluating the incidence of RVO based on the status of HTN treatment. Regarding ocular conditions, if the onset of RVO was earlier than 6 months before the first visit to our clinic, the patients were not considered to be in the acute phase and were thus excluded from the study. Also, patients with the following conditions were excluded: previous retinal vascular disease suggesting arteriosclerosis, including old RVO, retinal artery occlusion, ocular ischemic syndrome, retinal arterial macroaneurysm, ischemic optic neuropathy, or any systemic or ocular diseases that could secondarily influence retinal vessels, such as posterior uveitis, diabetic retinopathy, hypertension retinopathy, or systemic lupus erythematosus. These conditions could bias the patients’ characteristics, and some may cause secondary RVO. Finally, patients were required to be aged 40 years or older to exclude young RVO cases, which might have resulted from other unusual etiologies.

### Patient selection and statistical analysis

Sex and age are potential confounders in this study since WCH is frequently seen in women and older adults [10]. Thus, sex and age were matched between the RVO and non-RVO cases. As there were much more non-RVO cases than RVO cases, this matching was done automatically by selecting two control cases for each RVO case, minimizing the sum of the normalized squares of differences in age and sex between the RVO case and the controls.

We computed and compared basic demographics, including age, sex, the proportion of patients on HTN medication, and both systolic and diastolic office BP values. Continuous variables are presented as mean ± standard deviation. The Student’s t-test was used for continuous variables, while the chi-square test was employed for categorical variables. For the comparison of the proportion of sustained/uncontrolled HTN between the RVO and non-RVO groups, we used a chi-square test on the subset of patients with either normal office BP or sustained/uncontrolled HTN. To compare the proportion of WCH/WCUH between the RVO and non-RVO groups, the comparable analysis was conducted on the subset of patients with either normal office BP or WCH/WCUH. We also conducted a subgroup analysis on patients either not on HTN treatment or those undergoing HTN treatment. In each analysis, we calculated the odds ratio.

In detecting differences in the proportion of WCH/WCUH between the RVO and non-RVO patients, we found that 45 and 90 subjects, respectively, would be required, with expected proportions of 0.3 and 0.1, respectively, a power of 0.80, and an alpha of 0.05.

All data processing and analyses were performed using JMP Pro 17 (SAS institute).

## Results

**Fig. 1:**
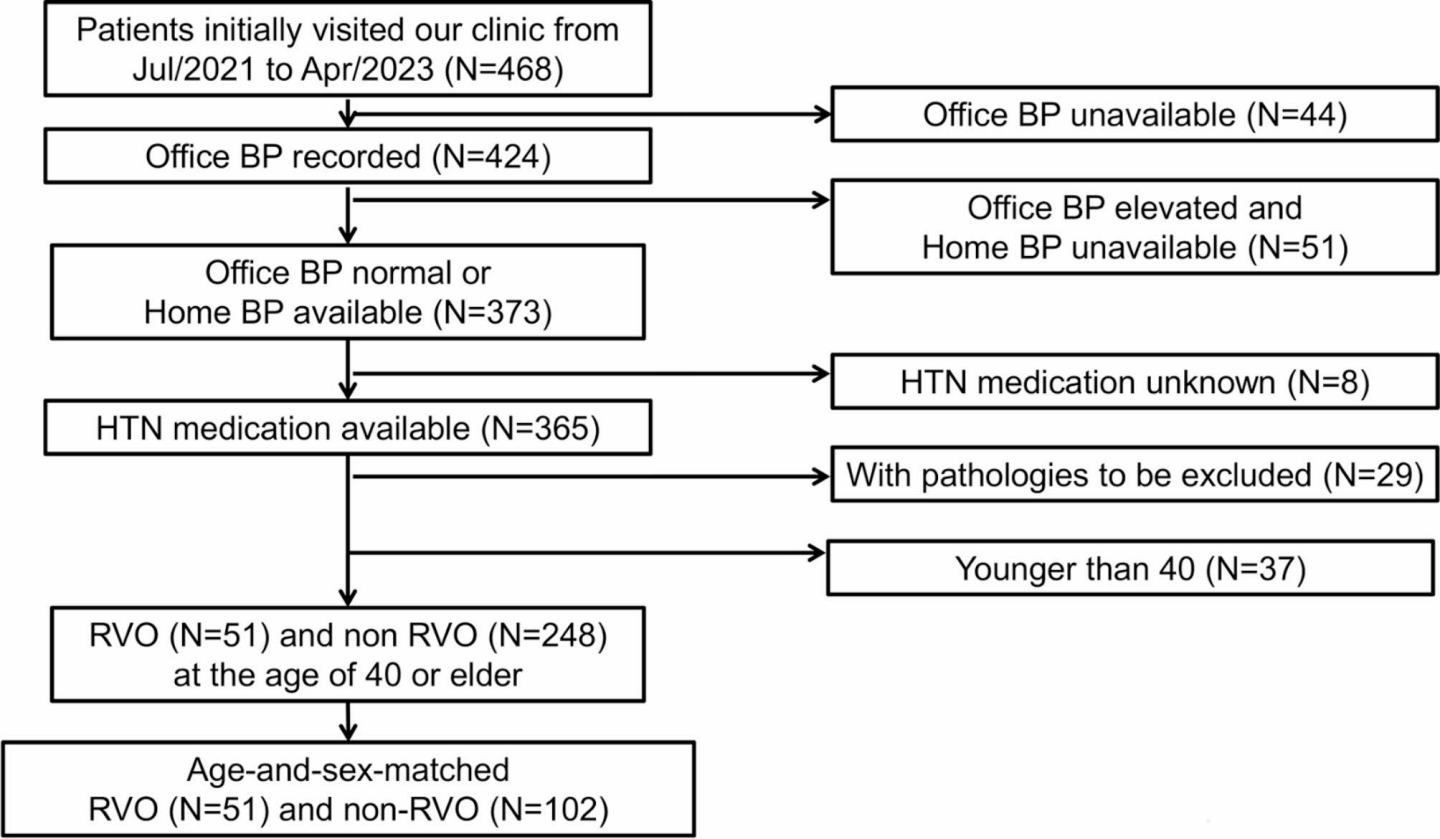
Patient selection. BP, blood pressure. HTN, hypertension. RVO, retinal vein occlusion

A total of 468 patients presented to the outpatient clinic during the study period. Patient selection was performed as illustrated in Fig. 1. After excluding the patients if office BP was unavailable, home BP unavailable under elevated office BP, HTN medication unknown, they had pathologies to be excluded, or were younger than 40, there were 51 RVO patients and 248 non-RVO patients. After age-and-sex matching, 51 RVO patients and 102 non-RVO patients were selected for analysis. Basic demographics of the population is shown in Table 1. Age, sex, and the proportion of patients with HTN treatment was similar between the groups (Student’s t test or chi-square test). The RVO group included 36 branch vein occlusion, 10 central retinal vein occlusion, and 5 hemi-central retinal vein occlusion. The non-RVO group included exudative age-related macular degeneration (*n* = 33), central serous chorioretinopathy (*n* = 17), epiretinal membrane (*n* = 10), retinal dystrophy (*n* = 9), dry age-related macular degeneration (*n* = 7), macular edema with posterior non-infectious uveitis (*n* = 7), pathological myopia (*n* = 4), macular hole (*n* = 2), tumor (*n* = 2), idiopathic macular telangiectasia type1 (*n* = 2), congenital macular anomaly (*n* = 1), and visual disturbance that was considered not associating with retinal/macular diseases (*n* = 8).

**Table 1:** Demographics of the patients included in the study. RVO, retinal vein occlusion. BVO, branch vein occlusion. CVO, central retinal vein occlusion. HCVO, hemi-central retinal vein occlusion

		RVO	Non-RVO	*P*-value
No. of cases		51	102	
Sex				
	Men (%)	21 (41)	42 (41)	
	Women (%)	30 (59)	60 (59)	1.00
Age, years				
	40–49	1	3	
	50–59	9	18	
	60–69	10	19	
	70–79	20	40	
	80–89	8	17	
	90 -	3	5	
	Average age	70.6 ± 11.7	70.5 ± 11.6	0.94
With HTN medication (%)		23 (45)	34 (33)	0.16
Diabetes		5 (10)	14 (14)	0.67
Hyperlipidemia		14 (27)	16 (16)	0.13
Type of RVO				
	BVO (%)	36 (71)		
	CVO (%)	10 (20)		
	HCVO (%)	5 (9)		

**Fig. 2:**
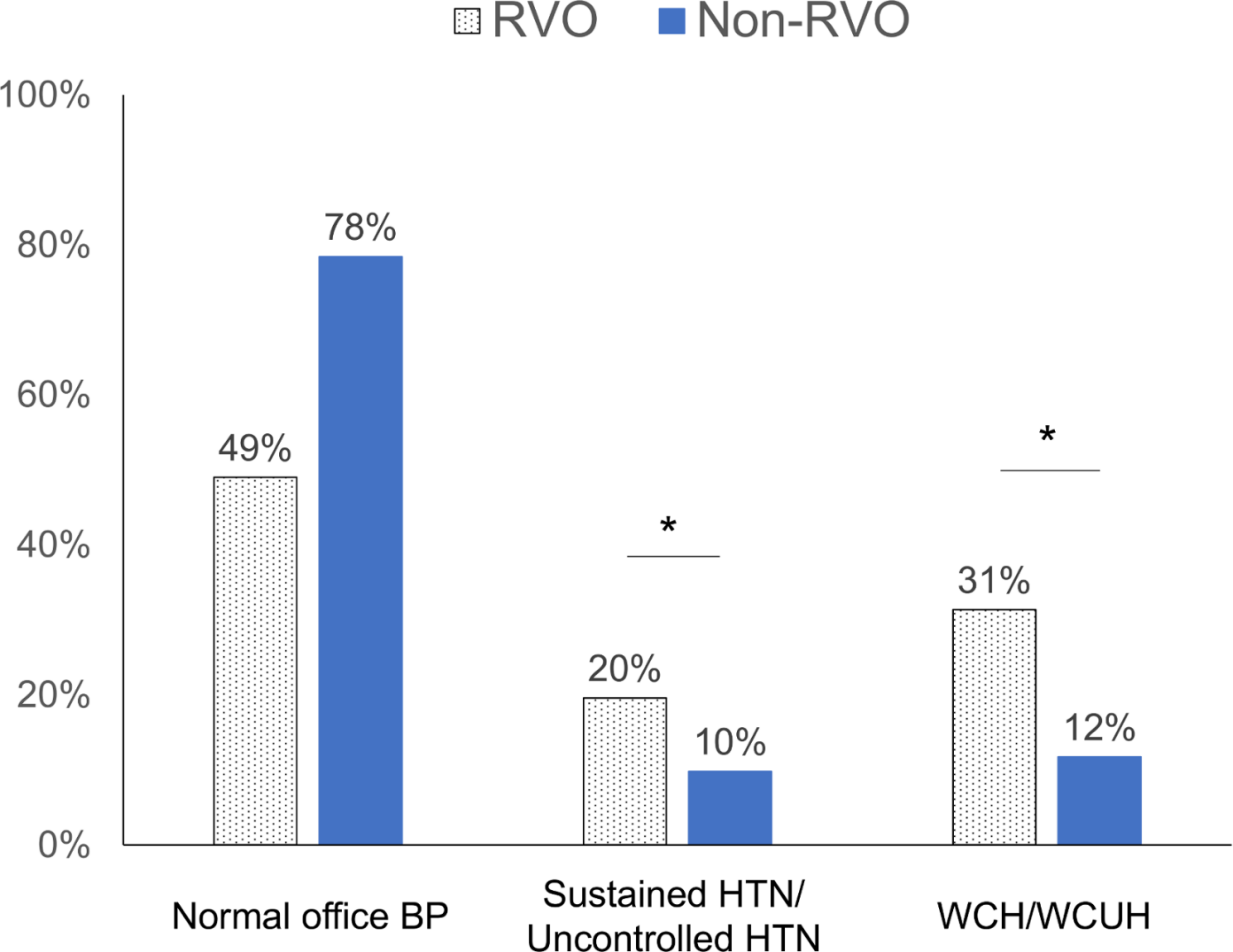
Proportion of the type of hypertension in the RVO or the non-RVO patients. *: *p* < 0.05, chi-square test. BP, blood pressure. HTN, hypertension. RVO, retinal vein occlusion. WCH, white coat hypertension. WCUH, white coat uncontrolled hypertension

Mean office BP values are presented in table 2. Both systolic and diastolic BP values were significantly higher in the RVO group than in the non-RVO group for all patients and for those not on HTN medication. For patients on HTN medication, these values trended higher in the RVO group compared to the non-RVO group, but the difference was not statistically significant. Among patients with elevated office BP, whether in the overall cohort, the RVO group, or the non-RVO group, the proportions with only elevated systolic BP were 75%, 73%, and 77% respectively. Those with only elevated diastolic BP accounted for 4%, 0%, and 2% respectively, while those with both elevated systolic and diastolic BP were 23, 22, and 23%, respectively. For those with elevated office BP, home BP values were also examined. Based on office/home BP measurements, patients were categorized as having normal office BP, sustained/uncontrolled HTN, or WCH/WCUH (as detailed in table 3). The RVO group had a significantly larger proportion of patients with persistent/uncontrolled HTN or WCH/WCUH compared to the non-RVO group (*p* = 0.02, odds ratio of 3.2 [95% CI: 1.2, 8.6], and *p* = 0.0007, odds ratio of 4.3 [1.8, 10.2], as shown in Fig. 2). In the subgroup not on HTN medication, the proportion of patients with persistent HTN or WCH in the RVO group was also significantly higher than in the non-RVO group (*p* = 0.04, 4.0 [1.02, 15.8], and *p* = 0.003, 5.1 [1.6, 16.2], respectively). Meanwhile, in the subgroup on HTN medication, the proportion of patients with uncontrolled HTN or WCUH in the RVO group showed a trend towards being higher compared to the non-RVO group, but the differences were not statistically significant (*p* = 0.28, 2.2 [0.5, 9.1], and *p* = 0.10, 3.1 [0.8, 11.8], respectively).

**Table 2:** Systolic or diastolic office blood pressure in RVO or non-RVO patients with or without HTN treatment. P values indicated were not corrected for multiple comparison. RVO, retinal vein occlusion. BP, blood pressure. HTN, hypertension

		RVO	Non-RVO	*P* value
Total	No. of Patients	51	102	
	Systolic office BP	140.5 ± 15.8	131.6 ± 15.1	0.0009
	Diastolic office BP	80.3 ± 11.9	73.8 ± 11.2	0.0013
HTN medication (-)	No. of Patients	28	68	
	Systolic office BP	140.1 ± 17.1	130.4 ± 15.7	0.009
	Diastolic office BP	81.5 ± 11.1	73.4 ± 11.3	0.002
HTN medication (+)	No. of Patients	23	34	
	Systolic office BP	141.0 ± 14.6	134.1 ± 13.6	0.07
	Diastolic office BP	78.7 ± 13.0	74.7 ± 11.1	0.2

**Table 3:** Number of each patient with or without RVO classified based on the office/home blood pressure and the hypertension medication. HTN, hypertension. RVO, retinal vein occlusion. WCH, white coat hypertension. WCUH, white coat uncontrolled hypertension

	HTN medication	RVO (*n* = 51)	Non-RVO (*n* = 102)	*P* value
Normal office BP	(-)	14 (27%)	56 (55%)	Not calculated
	(+)	11 (22%)	24 (24%)	Not calculated
	Total	25 (49%)	80 (78%)	Not calculated
Sustained HTN/ Uncontrolled HTN	(-)	5 (10%)	5 (5%)	0.04
	(+)	5 (10%)	5 (5%)	0.28
	Total	10 (20%)	10 (10%)	0.02
WCH/ WCUH	(-)	9 (18%)	7 (7%)	0.003
	(+)	7 (14%)	5 (5%)	0.1
	Total	16 (31%)	12 (12%)	0.0007

## Discussion

In the current study, we investigated the association of WCH/WCUH, as well as sustained/uncontrolled HTN, with RVO in a retrospective case-control study. The results showed that, similar to sustained/uncontrolled HTN, the proportion of WCH in the RVO group was significantly greater than in the non-RVO group. The subgroup analysis for patients without HTN medication also showed comparable results. In the subgroup with HTN treatments, however, the proportion of WCUH or that of uncontrolled HTN tended to be greater in the RVO group, but the difference was not statistically significant.

Risk factors for RVO include hypertension, hyperlipidemia, or diabetes, which require optimal management if found during the systemic workup for RVO patients [17–19]. This is especially the case with hypertension. Physicians are recommended to closely monitor blood pressure and consider initiation or modification of therapy once they find RVO in the eye of their patients [20].

Meanwhile, until recently, intervention for WCH has not been generally indicated due to the lack of the evidence suggesting WCH as a risk for increasing mortality or cardiovascular events [12–14]. Considering that medication for hypertension is based on home BP these days [6], it is possible that patients with WCH are not treated until they develop sustained hypertension. However, there is growing evidence suggesting the systemic influence of WCH. Recent studies have shown not only that WCH was the risk for developing sustained hypertension [21], but also that it increased CV risk or mortality [9, 22]. Moreover, sub-clinical organ damage including left ventricular hypertrophy and low estimated glomerular filtration rate (eGFR) indicating decreased renal function [15, 23–25], and thickening of carotid intima-media thickness (CIMT) [23, 26] has been reported to be associated with WCH. Brachial-ankle pulse wave velocity, indicator of artery stiffness, was also found to be increased in WCH patients [16]. These results imply that WCH could influence arteriolar hypertrophy or artery stiffness.

As for RVO, these subclinical vascular impairments such as left ventricular hypertrophy [27] or decreasing eGFR [28] have also been reported to be associated with RVO. Moreover, CIMT thickening [29] and pulse wave velocity (PWV) [29], both of which are indicators for artery stiffness, were also observed in RVO patients. The results of the current study indicate that patients with WCH might have a greater risk for developing RVO, at least partly, due to increased artery stiffness complicated with WCH.

In the subgroup analysis of the current study, the subgroup of patients with WCUH, who had HTN medication and normal home BP but elevated office BP, tended to be greater in RVO than in non-RVO, but there was no statistical significance. The lack of significance might be explained by two reasons: either an insufficient sample size to show significant difference or there being actually no significant difference. Recent meta-analysis showed that while untreated WCH was associated with an increased risk for cardiovascular events or mortality, WCUH or treated WCE was not significantly associated with these risks, supporting the latter possibility. Patients receiving treatment may be harmed by overly aggressive management [9]. As for RVO, Patients with WCUH could have an intrinsic risk for RVO because they were hypertensive, so it would be as difficult to elucidate whether white coat effect could be an additional risk for RVO to hypertension itself. Further studies with greater number of patients with WCUH or controlled HTN would be helpful to verify the hypothesis.

Our study had several limitations. First, it included a relatively small number of patients. A larger dataset should be used to confirm the results of this study. Second, the retrospective design of this study introduces potential inaccuracies and biases, such as missing or incomplete data. For instance, data on history of previous cardiovascular diseases, diabetes, hyperlipidemia, or smoking habits, which are known risk factor for arteriosclerotic diseases, may be recorded more often in RVO cases. Only incomplete information was available regarding the specific details of the treatment for HTN, which was not analyzed. While office BP measurements were routinely recorded, the home BP of patients with normal office BP was unavailable in most cases. As a result, we could not account for the possibility of masked HTN in these patients, which is a known risk factor for cardiovascular events and mortality. However, the aim of our study was to examine prevalence of WCH in RVO patients. Third, the home BP measurements that were available were not standardized, preventing us from using these values for quantitative analysis. A more detailed examination of the absolute differences between office and home BP would likely yield more detailed findings. Fourth, BP measurement at a single visit may not be sufficient for accurately diagnosing WCH. Typically, ambulatory pressure monitoring is better to confirm WCH [30], and our reliance on a single measurement could have affected the accuracy of our classification. Furthermore, the absence of 24-hour ambulatory BP monitoring is another limitation of our study. Such monitoring is more suitable for a thorough understanding of a patient’s BP profile over time and its relevance to morbidity. To further validate our findings, a prospective study design incorporating 24-hour ambulatory BP monitoring would be beneficial.

In conclusion, WCH was more commonly observed in patients with RVO than in those without RVO. Our results may help ophthalmologists address high BP detected in RVO patients. Although there are a large number of WCH patients [31], the management of WCH is still not standardized. Therefore, ophthalmologists need to clarify the impact of WCH/WCUH on RVO or other retinal vascular diseases and collaborate appropriately with internists for optimal assessment and management of systemic BP and ocular pathologies.

## Data Availability

No datasets were generated or analysed during the current study.
